# Accessibility and suitability of residential alcohol treatment for older adults: a mixed method study

**DOI:** 10.1186/s13011-018-0183-0

**Published:** 2018-12-13

**Authors:** Sarah Wadd, Maureen Dutton

**Affiliations:** Substance Misuse and Ageing Research Team, Tilda Goldberg Centre for Social Work and Social Care, University of Bedfordshire, University Square, Luton, LU1 3JU UK

**Keywords:** Alcohol - older people - older adults - elderly - rehab - residential rehabilitation - age discrimination, Treatment

## Abstract

**Background:**

Whilst alcohol misuse is decreasing amongst younger adults in many countries, it is increasing in older adults. Residential rehabilitation (rehab) is a vital component of the alcohol treatment system, particularly for those with relatively complex needs and entrenched alcohol problems. In this study, we sought to find out to what extent rehabs in England have upper age limits that exclude older adults, whether rehabs are responsive to older adults’ age-related needs and how older adults experience these services.

**Method:**

This is a mixed method study. A search was carried out of Public Health England’s online directory of rehabs to identify upper age thresholds. Semi-structured qualitative interviews were carried out with 16 individuals who had attended one of five residential rehabs in England and Wales since their 50th birthday. A researcher with experience of a later life alcohol problem conducted the interviews.

**Results:**

Of the 118 services listed on Public Health England’s online directory of rehabs, 75% stated that they had an upper age limit that would exclude older adults. Perceived differences in values, attitudes and behaviour between younger and older residents had an impact on older residents’ experience of rehab. Activities organised by the rehabs were often based on physical activity that some older adults found it difficult to take part in and this could create a sense of isolation. Some older adults felt unsafe in rehab and were bullied, intimidated and subjected to ageist language and attitudes.

**Conclusion:**

This study identified direct and indirect age discrimination in rehabs contrary to the law. Further research is required to find out if age discrimination exists in rehabs in other countries. Rehabs should remove arbitrary age limits and ensure that they are responsive to the needs of older adults.

## Background

In England, those aged 65–74 are the only age group where daily alcohol consumption is increasing [1, 2]. In Scotland, harmful, hazardous and binge drinking is increasing amongst those aged 65–74 but decreasing in other age groups [3]. In Wales, those aged 65 and over are the only age group where drinking above the daily guidelines is increasing [4]. In Northern Ireland, the most noticeable increases in alcohol consumption in recent years have been amongst those aged 60–75 [5]. Today, for the first time in recent history, drinkers aged 55–64 in England and Scotland drink more and are more likely to exceed the recommended weekly guidelines than any other age group [1, 3]. On average in England, weekly consumption is 17.2 units for men and 8.3 units for women aged 55–64 and in Scotland, 17.6 units for men and 8.0 units for women [1, 3]. A unit of alcohol is 8 g or 10 ml of alcohol. Alcohol consumption is also increasing in older adults in the United States [6] and other European countries [7]. The World Health Organisation has identified alcohol-related harm among older adults as an increasing concern [8].

Residential alcohol treatment services (rehabs) are a core component of the alcohol treatment system worldwide. In the United Kingdom (UK), they are provided by the voluntary and independent sector. The National Treatment Agency for Substance Misuse recognises that “residential rehabilitation is a vital and potent component of the drug and alcohol treatment system….anyone who needs it should have easy access to rehab” [9]. In 2015/2016, 812 people aged 50 and over and 73 people aged 65 and over received residential rehab for an alcohol problem in England [10]. The majority of rehabs in the UK accept people for both alcohol and drug problems. Unlike other residential health and social services such as care homes and inpatient mental health services which are generally segregated by age, rehabs can house residents from up to five generations who live alongside one another.

Rehabs in the UK and other countries vary considerably and are based on different models such as 12-step, therapeutic community and faith-based. Some rehabs provide residential detox facilities in addition to the rehabilitation programme. The feature that rehabs have in common is that residents have to live on-site for 24 h a day and are usually expected to be abstinent before they start the programme. They tend to cater for people with relatively complex needs and entrenched substance misuse histories [9, 11]. Residents are usually expected to participate in regular groupwork sessions with other residents, a range of tasks around the house (domestic duties or gardening for example) and to participate in group social activities. People usually stay for 3–12 months. Treatment focuses on life skills and the skills required to sustain an alcohol or drug-free lifestyle. Residential settings are considerably more expensive than non-residential alternatives although there is evidence that initial costs of residential treatment are to a large extent offset by reductions in subsequent health care and criminal justice costs [12]. Whilst some people in the UK pay for the entire cost of their treatment, most people receive at least a contribution from public funds. The amount that people have to pay themselves depends on their income including private or state pension, benefits and earnings from employment.

This study came about because a member of the research team who herself had experience of a later life alcohol problem, had spent time volunteering in residential rehabs in England. This experience led her to question whether rehabs are well suited for older adults. She had also witnessed older residents being bullied by younger residents. Previous research has also found that older adults can be bullied and intimidated by younger residents in rehabs [13] and that rehab staff can be reluctant to accept referrals for older adults [14].

There has been very little research about the experiences of older adults in rehabs despite their widespread use worldwide and a growing population of older adults with alcohol problems. A quantitative study of men in the United States [15] examined older mens’ (55 and over) satisfaction with a rehab for veterans compared to satisfaction amongst younger (ages 21–39) and middle-aged men (ages 40–54). They concluded that older men perceived the rehab somewhat more positively than middle-aged and younger men. Given the unique physical, emotional and cognitive features of ageing, it is possible that older adults respond differently to treatment than younger adults. Relatively few studies report outcomes based on age. The few that have, mostly suggest that outcomes for older adults are at least as good as for younger adults [15–17] but one study found that the oldest age group (aged 75–96 years) had the shortest stays in treatment and were the least likely to be treated successfully [18]. However, their experiences have not been explored qualitatively.

In the United States, there are a small number of residential rehabs specifically for older adults (age-specific programmes) (e.g. Caron^1^ and Hazelden Betty Ford^2^). These programmes are adapted to meet the needs of older residents, for example, they are accessible to wheelchair users, those with limited mobility, hearing loss, visual and cognitive impairment, take into account co-existing health issues such as diabetes and cardiac problems when developing a treatment plan, have age-appropriate physical activities such as movement therapy (music, stretching) and meditation, provide support for chronic pain and medication management. Group therapy focuses on age-specific issues such as bereavement and age-related loss and loneliness. A perceived benefit of age-specific programmes is the effects of social bonding with same-age peers [19]. Evaluation studies have shown that older adults are more adherent to treatment and have better outcomes in age-specific rehabs compared to mixed-age rehabs [19–23]. We are not aware of any rehabs specifically for older adults in the UK but rehabs specifically for young people do exist.

This study used interrogation of an online database of residential rehabs in England and semi-structured qualitative interviews with people aged 50 and over residing in rehabs to find out:-

1. To what extent do residential rehabs in England have upper age thresholds which exclude older adults?
2. Do older adults have age-related needs in rehabs?
3. How do older adults experience rehabs?

It was expected that some residential rehabs would have upper age thresholds and that mixed age rehabs might present challenges for older adults due to their lifestage and age-related needs.

## Methods

### Upper age thresholds in residential rehabs and disabled access

In July 2016, we carried out a search of ‘Rehab Online’ – http://www.rehab-online.org.uk/searchresults.aspx . This is Public Health England’s online directory of residential rehab services. Public Health England is an executive agency of the Department of Health and Social Care in the United Kingdom whose remit is to protect and improve the nation’s health and to address inequalities. Rehab Online is a comprehensive source of information on voluntary and private sector rehabs in England and is used by service users, commissioners, care managers and providers.

We used the directory’s “find a rehab” search facility and added the “alcohol treatment” filter to identify all rehabs which offer alcohol treatment. Each rehab in the directory has a “who we treat” tab which includes a category for age. We looked at this tab for each rehab and counted the number of rehabs with different age thresholds. It is rehab staff who are responsible for entering the age thresholds and other information in the database.

During the qualitative interviews, questions were raised about access to rehabs for people with disabilities or limited mobility. Therefore, whilst it was not one of our original objectives, we also used the “facilities/vacancies” tab to find out the level of wheelchair access which was categorised on the website as “none”, “limited” or “full”.

### Interviews with older residents

#### Interviewer

The interviewer was a female who had lived experience of a later life alcohol problem and had spent time volunteering in residential rehabs in England. She chose not to disclose her own experience of an alcohol problem to interview participants.

#### Data collection

Through our professional networks, we secured participation from five residential rehabs, four from England and one from Wales. We asked each participating service to invite 3–4 residents who had attended the rehab since their 50th birthday to take part in the study. Assurances were given that neither the services nor the residents would be identifiable from the outputs of the study.

Semi-structured qualitative interviews were carried out with 16 residents in the rehab premises between September 2016 and May 2017. Topics covered in the interviews were experience of rehab and living alongside younger residents, whether the individual felt that the needs and preferences of older adults were different to younger residents, whether these needs were met, whether the rehab could have done anything to make the service easier to access, more welcoming or suitable for older adults and whether the participant would have preferred to be in a residential rehab specifically for older adults.

The interviews lasted 30–60 min and participants were given a £10 gift voucher to thank them for their time. All interviews were tape recorded (with permission) and transcribed verbatim before being coded.

#### Data analysis

The coding and categorising of data followed the approach of developing an analytic hierarchy, that is, of moving from data management (generation of themes) to descriptive accounts (assigning meaning) to explanatory accounts (developing more abstract concepts) [24]. This began with the identification of first-level codes which were then grouped into categories and then synthesised within thematic domains. One member of the research team took the main responsibility for coding but a researcher with lived experience of a later-life alcohol problem cross-checked, verified and refined the codes and themes.

Of the 16 participants interviewed, 6 were women and 10 were men. Fifteen of the participants were in the residential rehab for an alcohol problem and one for a drug problem. Participants ranged in age from 52 to 73 years with an average (mean) age of 59 years. All names have been changed and other identifying information has been removed. We have not given participants exact ages in this paper to prevent deductive disclosure.

## Results

### Upper age thresholds in residential rehabs and disabled access

Of the 118 services listed on the Public Health England database for alcohol rehabs, excluding those specifically for young people (under the age of 18), three quarters (75%) stated that they had an upper age limit of anywhere between 50 and 90 years. By the time someone has reached the age of 66, more than half of the rehabs (55%) exclude them. The upper age thresholds are shown in Table 1.

**Table 1:** Upper age thresholds in residential rehabs listed on the Public Health England database for rehabs

Upper age threshold	No of rehabs	% of total^a^	Cumulative %
50 years	2	2	2
60 years	2	2	4
64 years	1	1	5
65 years	59	50	55
70 years	4	3	58
75 years	12	10	68
80 years	7	6	74
85 years	1	1	75
90 years	1	1	76
No upper age threshold	29	25	–
TOTAL	118	–	

^a^Does not add up to 100% due to rounding

We also searched the database for residential rehabs which stated that they had limited or no disabled access. Of the 118 services listed, 75% said they had limited or no disabled access.

### Interviews with older residents

The qualitative analysis identified four key themes.

#### The “generation gap”

Perceived differences in values, attitudes and behaviour between younger and older residents (one participant referred to this as “the generation gap”) had an impact on older residents’ experience of rehab. Participants compared the experience to “walking into a nursery school”, “living in a student house” and “being back at school”. They gave accounts of:-

- “Childish behaviour” – “stupid jokes and stupid comments and throwing cushions when there’s no need”
- Different interests and use of leisure time – “they’re sort of still into this mountain biking, boyish stuff and they’re in a group and they lay around sleeping on the settees”
- Aggressive/offensive language, comments and sexual innuendos – “they were using a different language, it was more aggressive in tone”; “the language is pretty bad, almost every other word is effing this and effing that”
- Different attitudes to treatment – “I think when people are laughing and joking and things like that and I’m thinking, no that’s just like a child, like play and things, you should be taking it really serious”
- Higher energy levels – “I tend to get tired quicker these days, their energy levels are bouncing around”

Some older residents felt unsafe in the rehab environment.

*“I came down at 6 o’clock in the morning to put my rubbish out and people have gone, opened the back door, gone outside for a cigarette and then left it open so it’s open for anyone to jump over the fence and walk in. People laugh about it but I think it’s very dangerous to leave the door open and knowing what sort of place this is as well, to hear that a couple of blokes have stolen from drug dealers and they think they’re going to come after them, I don’t want to hear that sort of thing and then somebody else is an ex-arsonist and it’s scary when you get older, you’re quite scared, you’re thinking what else is going to happen?”* **(Anne, late-fifties)**

Diversionary activities organised by the rehab were often based on physical activity such as mountain biking, caving, kayaking, football and hiking. Some older residents found it difficult to take part in physical activities with the younger residents and this could create a sense of isolation.

*“I can’t kick the ball in the garden [play football with other residents], that’s me walking away and being a lonely person which I’m used to….. I don’t feel like I belong, I don’t belong to being with them, playing or joking and laughing.”* **(Dan, mid-fifties)**

The fact that younger residents often had different interests presented challenges, but for some older residents, also an opportunity to try something new.

*“Dreadfully difficult when I started, “come on, let’s play Pictionary” on a Friday night, very difficult, “Let’s have a disco”, “let’s have a karaoke night”, “let’s go kayaking”, very difficult because all those things are, for older people, a lot of older people, outside their comfort zone….. I ended karaoke singing and kayaking and disco dancing and playing Pictionary and playing Bingo and joining in*.” **(Derek, early-seventies)**

*“It’s hard to talk to them because you don’t know what to say and then they think your being [pause] you do feel quite lonely at times because you can’t relate, the films they watch. Luckily we’ve got two lounges because it’s all boys’ films, horror and gore that sort of stuff so luckily the other television room we’ve got is more older, well I suppose over 30 basically, so we watch a different sort of television but there’s still that feeling of isolation, that you’re the oldest one here…there’ll be six blokes sitting there talking about, I don’t know, going to the gym or weightlifting and stuff happened, so and so from that film and you’ll be standing there with your tea thinking, oh God, now what do I do? Shall I go and sit with them? It’s like they’ve got their own little group and they’re talking about stuff I can’t even relate to.”* **(Anne, late-fifties)**

Even those residents who were generally very happy living alongside younger residents enjoyed some respite when the younger residents weren’t there.

*“Sunday afternoons are great because during the summer and autumn all the young people used to go out on a walk and go and play football in the park, then the house would just calm right down and you’d find people, average age old, were sitting reading the Sunday paper, that kind of thing. All of a sudden, bang, they would come back, the papers would be everywhere. It’s like living with a bunch of puppies to be honest, but that’s what happens I suppose if you take people who are young, fit, rehabilitating and you put them into an enclosed space.”* **(Darren, mid-sixties)**

### Age prejudice

Participants in this study experienced age prejudice. Younger residents sometimes called them names such as “old fella” and “granddad”. Participants described instances where younger residents and staff expressed ageist attitudes.

*“A guy from Liverpool [resident] said it ain’t worth it, recovery at your age”.* **(Derek, early- seventies)**

*“What they [workers in rehab] do say is you’re looking too high, your goals are too high for your age group”.* **(Bob, early-fifties)**

Some older residents had experienced intimidation and threats of violence from younger residents but it was not clear whether they were targeted specifically because of their age.

*“There were three guys threatened to kill me…..I said “I tell you what, I’ll get a knife, I’m not sharpening it for you, and you can cut my throat”…..I called their bluff and they didn’t do it. They used to shove notes under my door and all this, put my glasses in a doggie bag somewhere….They shoved them [the glasses] somewhere, they hid them and I had to try and find them.”* **(Scott, early-seventies)**

It was clear that some of the residents experienced ‘felt stigma’ because of their age. They used ageist terms such as “old fart”, “miserable old bat” and “fuddy duddy” to talk about themselves or the way that they thought younger residents viewed them. Older residents themselves had stereotypical ideas about older adults; “I think older people can be a bit grandiose”, “older people are a bit miserable”, “[older people are] stuck in their ways”, “if you just had a whole bunch of older people, the place would smell of wee and cabbage”. Some used ageist terms to refer to younger residents such as “childish”, “juvenile” and “babyish” and described younger adults as “intimidating”, “selfish”, “lazy” and “[requiring to be] almost looked after”.

A number of participants expressed surprise that they had been offered a place in the residential rehab, ‘despite their age’.

*They look at people of my age, “no point” “they’re more likely to put the funding to someone who’s younger.”….I think they think you’re a bit of a ‘spent penny’ at a certain sort of age*. **(Bob, early-fifties)**

Relationships are crucial when it comes to community-oriented residential services. Some participants felt that their age resulted in social rejection whilst others were not aware of divisions and did not feel excluded.

*“I found it hard to be accepted by the younger people but similarly, the younger people didn’t want to be accepted by me….Even though I was open to being approached, they didn’t want to approach me because of the fact that they thought I was old and I wouldn’t understand their problems. That is what they have said rather than my impression of what they would say.”* **(Darren, mid-sixties)**

*“You never get that feeling of the older ones are sitting here and the younger ones are sitting there because you all blend together and it was the same in detox, because you’re all there for the same reason so it doesn’t matter how old you are, you’ve all got a connection and so there’s no them and us.”* **(Mark, mid-fifties)**

A number of participants were keen to point out that they felt supported by younger residents.

*“Don’t get me wrong, they’re young at the end of the day, but they’re polite, it’s just their age. Say if you were struggling with something, they’d take it straight off you, “I’ll carry it upstairs for you”, they’re really good like that.”* **(Julie, early-fifties)**

### Autonomy, privacy and space

One of the features of rehab that participants struggled with was lack of autonomy. This can be difficult for people of any age, but some of the participants felt this was particularly challenging for older residents.

*“There’s lots of rules and regulations and they’re all meant to be there for my care but I find it quite difficult because I feel like I’m a grown-up person who’s been in charge of my life for a long time and I find it quite difficult not to go to the shops and not do this and not do that…. I’m nearly 60 and I can’t go the shops.”* **(Sarah, late-fifties)**

Another issue that participants struggled with was sharing bedrooms with younger residents.

*“I haven’t shared living accommodation with anyone except my wife and family for 40 years. I’ve come into shared accommodation and I was in a shared bedroom with a 26-year-old. The 26-year-old, it was like living with a chinchilla. They were everywhere, bounding around. They didn’t go to bed until two o’clock in the morning. I got up in the morning, I pottered around, they were still in bed. Literally on a number of occasions I turned the mattress so they’d get out of bed.”* **(Darren, mid-sixties)**

However, some older residents had successfully shared a room with younger residents.

*“Somebody new came in [to share bedroom] and he’s younger than me and we got on really well, every now and again you want to go to bed early because you want time on your own but yes, you’d like a single room with your own bathroom and everything like that but as I said, you’ve got to think about where you are.”* **(Mark, mid-fifties)**

It was important to older residents that they had a place to “retreat” or “take solitude” – one participant said he needed a “bolt-hole”.

*“It would be nice to have a quiet space it would be nice to have the option, even, of not even single occupancy but having the option of spending time with people of my own age occasionally. It’s nice, it’s lovely being with younger people, I like being with younger people, as I said I was a [profession that involves working with children]. It’s nice to get their ideas, but sometimes it is just a little bit wearing. I feel like the old fuddy duddy that I’m sure they believe I am.”* **(Darren, mid-sixties)**

In some residential rehabs, spending time on your own was described as “isolating” and actively discouraged by staff. This was frustrating to those who sought time and space alone.

### Mixed-age versus age-specific rehabs

Given some of the tensions between older and younger residents identified in this study, we wanted to find out whether participants would prefer to be in a residential rehab specifically for older adults.

Some residents felt that they would have preferred an age-specific service.

*“I’d feel a lot more comfortable [with people of own age] and you’d have something more in common, you can talk to each other about different things.”* **(Anne, late-fifties)**

However, some residents felt differently. They embraced the intergenerational social environment in the rehab.

*“Some of us, we come in here, we can’t remember the last time we’ve laughed and the youngsters, they’re brilliant and the kindness and empathy they have, genuine kids. I wouldn’t want to be in a treatment centre full of people my age, no way, no way, I’d probably come out feeling 90!”* **(Karen, mid-fifties)**

*“If you said, look, there’s a rehab centre and it’s for people over 50, I’d have run a bloody mile. Why the hell would you create an environment like that? It’s stigmatising, you know? You are over 50, therefore you are special and different and therefore we’re going to make you special and different? That’s not a good environment for recovery, you’re just like everybody else.”* **(Derek, early- seventies)**

For some, being in a rehab with younger adults provided them with an opportunity to pass on their wisdom and experience to younger residents, a role that was described as “being the elder statesman” and a “father role”. There was also a recognition that older residents had something to learn from younger residents.

*“I think you need the younger people and the older people to be there because you need the breadth of experience that each of them can bring to the general melting pot. You need the mixture of social backgrounds, you need the mixture of addiction types….The mixture of ages is very important. I can see that some people would love to have only people over 50 and I’m sure it would make them feel safe but I think they would lose a lot. Young people have an awful lot to tell us, if we just listen to the right bits….I think it would be a poorer programme without the mixture of ages to be honest.”* **(Darren, mid-sixties)**

Some of the participants felt that having a shared experience of addiction was a great leveller that bound them together regardless of age and generational differences.

*“The thing with being in addiction is because you’re all the same, it doesn’t matter what you’re addicted to, it creates a bond anyway, regardless of age or circumstances.”* **(Clare, late-sixties)**

Some participants felt that rehabs could do more to meet the needs of all ages.

*“I think that the service provider should be aware of the different requirements of the age groups and try and facilitate those better and be more prepared for them rather than just saying, anyone from the age of 21 to 70 is a client. Thinking that they will have the same requirements and will require the same services, they don’t obviously because of their age. There are age-specific requirements, as you get older, you need different stuff.”* **(Darren, mid-sixties)**

## Discussion

This study has found that older adults are excluded from three quarters of residential rehabs listed on Public Health England’s online directory of rehabs on the basis of their age. In response to our finding, Public Health England has removed the option for rehabs to enter an upper-age threshold for their service in the directory. Whilst it was beyond the scope of this study to explore why residential rehabs are imposing these age limits, conversations with service managers suggests this is due to an assumption that the care needs of an older adult will be higher and that their care needs cannot be met in a rehab. However, age alone cannot determine care needs. It is quite possible that the care needs of a 40-year-old will be higher than those of a 65-year-old.

Age discrimination can either be direct or indirect. Direct age discrimination occurs when people of comparable needs are treated differently or denied access to services purely on the basis of their age. By imposing age limits, residential rehabs are directly discriminating against older adults. Unjustifiable age discrimination is contrary to the Equality Act of Great Britain [25] which places a duty on services not to discriminate on age grounds. This discriminatory practice has no place in the substance misuse treatment system; a person’s access to rehab should be based on their individual condition and circumstances, not assumptions based on their age. Other research suggests prejudicial attitudes towards older adults with alcohol problems because of their age may be commonplace. A recent survey of health and social care practitioners in the UK found that participants had observed older adults being “written off” as too old to change, a perception that it is not worth intervening with older adults due to their potential life expectancy, older adults not referred to alcohol treatment, younger adults prioritised over older adults in terms of alcohol treatment and older adults guided into non-specialist services as a result of their alcohol problem (e.g. care home rather than rehab or older adults social work teams instead of community alcohol service) [26]. In 2009, a Healthcare Commission report found that people over 65 in England are often denied access to the full range of mental health services available to younger adults including alcohol and drug services and identified tackling age discrimination as a key priority for action [27].

Older residents who do become residents in residential rehab are likely to be a select population. They have overcome the barriers to access described above and are likely to be relatively amenable to living alongside younger residents in a community-oriented environment because they chose to enter the rehab knowing that they would be part of an intergenerational community. Even so, our interviews with older residents show that some found that living ‘cheek-by-jowl’ with younger residents and sharing domestic duties, social spaces, bedroom and bathroom facilities, can create tensions. Some older residents experience social exclusion, bullying and intimidation, felt unsafe and unable to participate in physical social activities with younger residents leading to further social exclusion. Older and younger residents held negative age stereotypes about one another and made disparaging remarks about members of other (and their own) generations.

Whilst intergenerational conflict did occur, there were also examples of intergenerational cohesion. Some older residents not only enjoyed the company of younger residents, they felt that their experience of rehab was enriched by it. They experienced kindness and compassion from younger adults. Being in an intergenerational rehab provided an opportunity to pass on their wisdom to younger residents and to take part in activities that they wouldn’t have participated in under normal circumstances. A number of participants felt that age was not important because members of the community were bound by their shared experience of addiction.

Services may indirectly discriminate against older adults even when, in theory, there is no obstruction to their access. Indirect age discrimination occurs when people from different age groups, with different needs, are treated in a similar way, with the result that the needs of older adults are not fully met. This is sometimes described as being ‘age-blind’.

Our findings suggest that some residential rehabs may not be sufficiently responsive to the needs of older adults. It would be simplistic to suggest that all older adults have the same needs based on a particular age categorisation. Generational groups are not homogenous units with predictable needs, preferences and behaviours. However, this study has identified some factors which, if implemented, would make rehabs more accessible and acceptable to older adults. These are discussed in the conclusions and recommendations below. More broadly, we suggest that residential rehabs strive to become ‘age-advantaged’. Age-advantaged means promoting policies and practices that increase cooperation, interaction, and exchange between people of different generations enabling all ages to share their talents and resources and support each other [28]. This intergenerational approach recognises that generational differences and similarities are a valid, important and enriching form of diversity that should be amplified and harnessed [29].

Whilst intergenerational rehabs can work well for some people and result in a melding of views and experience, some older adults are likely to benefit from being grouped with residents of a similar age. This could be done by similar units within a single residence or through different facilities.

This study has also found that the majority (75%) of residential rehab facilities report limited or no wheelchair access, indicating that physical accessibility issues may be a further barrier for older (and younger) adults who are disabled or have limited mobility. The reported levels of accessibility for people with disabilities may underestimate actual accessibility. Voss et al. found that substance misuse treatment providers in the United States frequently overestimate the accessibility of their facilities [30].

This study has limitations. In the qualitative element of the study, this primarily relates to the small sample size and the use of gatekeepers to identity a convenience sample which means that those who are most positive about the services may have been invited to take part in the study. We asked gatekeepers to select people with a variety of experiences of residing in a rehab to reduce this bias. The interviewer herself had experience of a later-life alcohol and had volunteered, but not resided in, a rehab. Some have cautioned that engaging peer interviewers can reduce objectivity; peer interviewers may lack distance and detachment and be more likely to take things for granted or assume that their experience is far more widespread than it is [31, 32]. On the other hand, peer-interviewers have the potential to discover knowledge that may otherwise go unnoticed by researchers without lived experience. The search of the online directory of rehabs to identify upper age limits is limited in that we cannot guarantee that all rehabs in England were listed. It is the responsibility of the services to register on the directory. However, it is unlikely that those rehabs in the directory will be different in terms of imposing upper age thresholds to services not registered in the directory.

## Conclusions and recommendations

This study has identified direct and indirect age discrimination in rehabs. In the worst case scenario, if this prevents older adults obtaining the treatment they require for their alcohol problem, age-discrimination may lead to preventable deaths. It is not clear whether these findings are generalisable to other countries but given the fact that age discrimination is prevalent across continents and cultures, this may well be the case.

Age discrimination in rehabs is a priority that requires more than superficial attention and a piecemeal approach. Rehabs should remove arbitrary age limits. A person’s access to rehab should be based on their individual condition, circumstances and ability to benefit not assumptions based on their age. Rehabs should develop an equality and diversity strategy and carry out an equality impact assessment. They should ensure that intergenerational awareness, skills and strategies are components of staff competency and encourage residents to invest time discovering what they share with residents from other generations such as needs, goals, interests and points of view. House rules for residents should make it clear that they should avoid discriminatory language, behaviour and ostracising those with protected characteristics (including age) identified in the Equality Act 2010 or equivalent legislation in other countries. On admission, staff should assess individual’s compatibility with existing residents and any risks due to challenging behaviour. Where risks are identified, plans should be put in place to support the individual to prevent and reduce risk. To promote autonomy, privacy and space, rehabs should provide single rooms with en suite bathrooms wherever possible and a variety of spaces/lounges where residents can have privacy and solitude. Staff should provide social activities that people of any age and level of fitness or mobility can enjoy. Management should consider imposing more flexible house rules and fewer, less physical housekeeping duties and ensure inclusion of people of all ages in the design, planning, delivery and evaluation of the service. Rehabs should consider developing units within existing rehabs or separate facilities specifically for older adults who are likely to benefit from or prefer an age-specific service.

## Abbreviations

Rehab: rehabilitation
UK: United Kingdom

## Footnotes

1
https://www.caron.org/our-programs/inpatient-treatment/seniors

2
http://www.hazeldenbettyford.org/treatment/models/specialized-programs/older-adults
